# Patient-derived tumour xenografts for breast cancer drug discovery

**DOI:** 10.1530/ERC-16-0251

**Published:** 2016-11-11

**Authors:** John W Cassidy, Ankita S Batra, Wendy Greenwood, Alejandra Bruna

**Affiliations:** 1 Breast Cancer Functional GenomicsCRUK Cambridge Research Institute, Li Ka Shing Centre, University of Cambridge, Cambridge, UK; 2 Department of OncologyUniversity of Cambridge, Cambridge, UK

**Keywords:** breast cancer, drug discovery, biomarker discovery, high-throughput screening, patient-derived tumour xenografts, targeted therapies, pharmacogenomics

## Abstract

Despite remarkable advances in our understanding of the drivers of human malignancies, new targeted therapies often fail to show sufficient efficacy in clinical trials. Indeed, the cost of bringing a new agent to market has risen substantially in the last several decades, in part fuelled by extensive reliance on preclinical models that fail to accurately reflect tumour heterogeneity. To halt unsustainable rates of attrition in the drug discovery process, we must develop a new generation of preclinical models capable of reflecting the heterogeneity of varying degrees of complexity found in human cancers. Patient-derived tumour xenograft (PDTX) models prevail as arguably the most powerful in this regard because they capture cancer’s heterogeneous nature. Herein, we review current breast cancer models and their use in the drug discovery process, before discussing best practices for developing a highly annotated cohort of PDTX models. We describe the importance of extensive multidimensional molecular and functional characterisation of models and combination drug–drug screens to identify complex biomarkers of drug resistance and response. We reflect on our own experiences and propose the use of a cost-effective intermediate pharmacogenomic platform (the PDTX-PDTC platform) for breast cancer drug and biomarker discovery. We discuss the limitations and unanswered questions of PDTX models; yet, still strongly envision that their use in basic and translational research will dramatically change our understanding of breast cancer biology and how to more effectively treat it.

## Introduction

Breast cancer (herein BC) is not a single disease, but is instead a collection of diseases that have distinct histopathological features and genetic and genomic variability linked to diverse prognostic outcomes. Recent research has highlighted this heterogeneity and defined ten molecular subtypes based on copy number and gene expression data from over 2000 patient tumours (Curtis *et al.* 2012). Coupled with advances in our understanding of intertumour heterogeneity, large scale genomics projects such as The Cancer Genome Atlas (Chang *et al.* 2013) and METABRIC (Pereira *et al.* 2016) have led to unprecedented annotation of the drivers of BC. It is hoped that these advances will help improve patient stratification for targeted therapy based on the molecular underpinnings of individual cancer samples, paving the way towards personalised cancer treatment. However, despite the remarkable success of many such targeted agents, most investigational agents fail to show significant efficacy in clinical trials. Consequently, the oncological drug space suffers from 88% attrition between Phase I agents and market approval (Hutchinson & Kirk 2011). In cases where agents are initially efficacious, responses can be fleeting and the development of drug resistance is often seen as an inevitable consequence of cancer’s heterogeneity (Aparicio & Caldas 2013). Our reliance on preclinical models, unable to reflect this heterogeneity is therefore likely to underpin failures of the drug development framework (Cassidy *et al.* 2015).

Realising these limitations, the scientific community has been driven to create novel preclinical models that are able to recapitulate the complexity of human cancers. Many have turned to patient-derived tumour xenografts (PDTXs) (Whittle *et al.* 2015), which retain the complex heterogeneity of their originating tumour samples (DeRose *et al.* 2011, Cassidy *et al.* 2015, Eirew *et al.* 2015). PDTX models of BC resemble primary tumours across the genomic, epigenomic and transcriptomic landscape and are stable across multiple passages (Marangoni *et al.* 2007, Kabos *et al.* 2012, Eirew *et al.* 2015, Bruna *et al.* 2016). As preclinical models, PDTXs can be used to predict clinical trial responses (Gao *et al*. 2015); however, there use in the discovery phase itself has thus far been limited.

The drug discovery process is almost exclusively split between rational design, based on structural biology of the target protein and high-throughput screening (HTS). HTS strategies typically rely on increasingly simplified biological models, such as cancer cell lines containing reporter constructs for the pathway of interest and highly complex compound libraries (Kenny *et al.* 2015). Although PDTX models are undoubtedly more biologically relevant than cancer cell lines, they are limited by low throughput and high establishment costs (Siolas & Hannon 2013, Whittle *et al.* 2015).

Herein, we discuss the role of PDTX models in the BC drug discovery process. We begin by considering currently available models of BC and their uses in the drug discovery process before presenting the argument for increased use of models accurately reflecting the complexity of human malignancies. This complexity brings specific considerations, particularly in the need for high-throughput drug combination screens and deep genomic characterisation of models to enable biomarker discovery. We conclude by reflecting on our own experiences in developing an integrated pharmacogenomic pipeline for breast cancer drug discovery using PDTX cells (or PDTCs).

## Preclinical models of breast cancer

BC is a collection of diseases with distinct biological traits and clinical outcomes. Thus, no individual model would be expected to completely recapitulate human BC in its entirety. Nevertheless, multiple models of BC have been established over the years, both patient derived and artificially engineered. For a full overview of these models, including their respective limitations, the reader is directed to an excellent review by Vargo-Gogola and Rosen (Vargo-gogola & Rosen 2007). In this section, we consider the models most often used in basic research and how these have fared in the drug discovery process.

### Breast cancer cell lines

BC cell lines have found extensive use in the investigation of proliferation, apoptosis, migration and the tumour-initiating cell (TIC) phenomenon. The first BC cell line capable of surviving in culture for longer than 2 months was isolated in Detroit in 1970 and named MCF-7 (Soule *et al.* 1973). This oestrogen receptor alpha (ER)-positive luminal cell line has been heavily relied on in the study of tamoxifen resistance, leading to predictive biomarkers of resistance in patients (Ross-innes *et al.* 2012). Together, MDA-MB-231 (a triple-negative cell line), T-47D (a luminal cell line) and MCF-7 account for more than two-thirds of all abstracts mentioning BC cell lines (Lacroix & Leclercq 2004). Experiments in cell lines were crucial in the development of one of the first targeted therapeutic agents launched in 1998 – the anti-HER2 Herceptin, a humanised antibody that binds to the ectodomain of HER2 (Carter *et al.* 1992), has demonstrated a remarkable clinical impact on HER2-positive BC. Cell lines have also helped to elucidate the mechanisms of primary and acquired resistance to Herceptin and are still being used for a significant proportion of BC research today. These early successes supported the use of cancer cell lines for both drug development and biomarker discovery (Heiser *et al.* 2012).

The artificial 2D system of *in vitro* culture has many drawbacks, and several attempts have been made to increase the relevance of these incredibly tractable models. A seminal paper published in 2003 by Al-Hajj and coworkers demonstrated the presence of TICs in pleural effusions from BC patients, which later were shown to be maintained in suspension as 3D spheroids called mammospheres (Al-Hajj *et al.* 2003, Dontu *et al.* 2003). Accumulating evidence has supported the use of this system to better understand the biology of specific facets of BC, drug resistance and metastasis (Reya *et al.* 2001, Polyak 2002, Wicha *et al.* 2006). Mammosphere cultures have also been used to unravel molecular mechanisms of signalling networks, for example, those underlying the apparently paradoxical role of transforming growth factor beta (TGFβ) in BC (Bruna *et al.* 2012). The authors further identified, using these 3D mammosphere cultures, that TGFβ BC subtype’s specific regulatory networks are dictated by epigenomic landscapes (Tufegdzic-Vidakovic *et al.* 2015). However, as yet, no significant progress has been made in drug discovery using these, or other, complex 3D culture systems.

The tumour microenvironment (TME), comprising the extracellular matrix (ECM) and stromal and immune infiltrates, has significant bearing on the course of tumour development (Straussman *et al.* 2012, Junttila & de Sauvage 2013). Bissell and coworkers pioneered methods to model the microenvironment in 3D-cultured BC cell lines (Lee *et al.* 2007). By profiling gene expression patterns, Kenny and coworkers were able to show that a panel of 27 BC cell lines more accurately reflected human tumours when cultured on recombinant basement membrane (rBM) (Kenny *et al.* 2007), though cells *in vivo* are subject to a plethora of ECM-derived signalling gradients not easily recapitulated *in vitro* (Cassidy 2014). The growth of BC cell lines as xenografts allows investigation of the tumour–stromal interactions seen *in vivo*. For example, Kitamura and coworkers have recently reported a CCL2-induced chemokine cascade that promotes metastasis to the lung in tail-vein-injected murine cell line E0771-LG (derived from a spontaneous medullary breast adenocarcinoma of C57BL/6 background) through the recruitment of metastasis-associated macrophages (Kitamura *et al.* 2015). By including stromal and immune components, such syngeneic cell-line xenografts can have substantial utility as preclinical models and in the drug development process.

### Breast cancer mouse models

Despite not always exhibiting typical histopathological phenotypes seen in human BC, genetically engineered mouse (GEM) models have been used extensively to investigate tumour initiation and progression. GEM models generally fall into three distinct histopathological categories: those closely resembling non-GEM tumours, those with unique transgene-specific phenotypes and those that resemble human malignancies (Cardiff *et al.* 2000). The choice of gene promoter and the mechanism of induction greatly influence the histological phenotype of the resulting tumour, and this needs to be taken into consideration for all GEM studies (Cardiff *et al.* 2000).

GEM models driven by the mouse mammary tumour virus (MMTV) promoter were used to characterise the effects of several now widely accepted oncogenes and tumour suppressors in BC (including tumour suppressors *Pten, Brca1* and *Trp53* and oncogenes *Erbb2, Myc* and *Ccnd1*) (Vargo-gogola & Rosen 2007). When combined with advanced intravital imaging, GEM models have also been used to elucidate the precise role of macrophages in BC metastasis. Jeffrey Pollard’s Lab has relied heavily on these models to show that the purported metastasis-associated macrophages are active promoters of the metastatic cascade rather than bystanders (Wyckoff *et al.* 2007). Like syngeneic models of BC, GEM models have the advantage of including native stromal compartments of a malignancy. In this regard, they may outperform many patient-derived models (see the ‘Strategies for breast cancer drug discovery’ section below) in the drug discovery process. However, a major limitation of GEM models is their tendency to form ER tumours suggesting that specific drug discovery processes are more suitable for other platforms (Medina & Thompson 2000).

Heterogeneity within a clonally expanding tumour is a consequence of intertumour heterogeneity (intrinsic molecular and cellular load) and clonal evolution upon selective pressures, which may also occur in spatially distinct tumour compartments. A recent study has uncovered a network of inter-clonal cooperation maintaining intratumour heterogeneity in a Wnt-driven MMTV GEM BC model (Cleary *et al.* 2014). The authors simulated targeted therapy by removing Wnt1 and found that relapsing basal populations recruited heterologous Wnt-producing luminal cells to restore cooperation. Alternatively, tumours evolved to rescue Wnt pathway activation through some other mutational event. In each case, drug resistance occurred from the cancer cell autonomous compartment in a fashion that could not be predicted easily. If such inter-clonal cooperation exists in human BC, this would underline the need for polyclonal preclinical models. However, it is worth noting that Wnt is thought to play relatively minor role in human BC (Curtis *et al.* 2012).

### *In vitro* patient-derived models of breast cancer

Realising the importance of the cancer cell autonomous compartment in driving therapeutic responses, many researchers have turned to organoid cultures to study a wide variety of processes involved in the development and disease. Beginning in 2009, the Clevers lab showed that single Lgr5+ intestinal stem cells (ISCs) could build crypt-villus structures *in vitro* without a supporting mesenchymal niche (Sato *et al.* 2009). Subsequent research by this lab has identified culture conditions for normal and malignant pancreatic (Huch *et al.* 2013*a*) and liver (Huch *et al.* 2013*b*) organoids, amongst other tissue types. Organoids are generally genomically stable over long-term passage (Huch *et al.* 2015), though it is unclear whether mixed organoid cultures of primary tumours can truly recapitulate the complex clonal heterogeneity seen *in vivo*.

Recently, a biobank of 20 human colorectal carcinoma (CRC) organoids was established and characterised by exome sequencing, RNA expression analysis and high-throughput drug screening (van de Wetering *et al.* 2015). The authors show that CRC organoids largely recapitulate most features of the originating tumour sample, and the biobank captures most of the mutational and expression landscapes observed in large CRC studies. The authors screened these cultures using an 83 compound library to identify the molecular signatures associated with drug responses (van de Wetering *et al.* 2015). Although this study represented an important step forward for the field, cancers exist as communities of competing and cooperating clones surrounded by infiltrating stromal and soluble growth factors (Tabassum & Polyak 2015), each of which contributes to intratumour heterogeneity and therefore treatment response. It is therefore likely that *in vitro* co-culture techniques would have to be of exquisite complexity to maintain cancer cells in a niche as suitable as that found in a murine host.

### Patient-derived tumour xenografts

Arguably the model best reflecting the complexity of human malignancies is the patient-derived tumour xenograft (PDTX). In this model, BC clinical samples are implanted and propagated in highly immunodeficient mice, typically NSG (NOD.Cg-*Prkdc^scid^* *IL2rg^tm1Wjl^*/SzJ) or NRG (NOD.Cg-*Rag1^tm1Mom^* *IL2rg^tm1Wjl^*/SzJ) strains. Early BC PDTX studies suffered from low transplantation efficiencies and consequently a limited diversity of models (Vargo-gogola & Rosen 2007, Siolas & Hannon 2013); for example, one study reported only three ER+ models in a cohort of 32 stably transplantable PDTXs (Zhang *et al.* 2013). Clearly, to be useful as preclinical models, the early bias towards aggressive triple-negative BCs (TNBCs) has to be overcome. In this context, a new protocol involving intraductal injection of cells in female mice has been developed with the hope to dramatically increase engraftment rates especially in less-aggressive tumour samples (Sflomos *et al.* 2016).

PDTXs reflect the originating sample’s morphological and molecular features, and these remained stable throughout serial passaging (Bergamaschi *et al.* 2009, DeRose *et al.* 2011, Bruna *et al.* 2016). Remarkably, PDTXs are also a community of clones of varying degrees of complexity to that found in the clinical population (Eirew *et al.* 2015, Bruna *et al.* 2016). Moreover, our recent observations also showed that most of the clonal composition of a given BC-originating sample prevailed in the matched PDTX.

The most obvious limitation of the PDTX model is the lack of a functional immune system. Recent studies have highlighted both the essential role of the immune system in tumour progression (Gajewski *et al.* 2013) and the utility of targeted therapies designed to activate immune components (Gubin *et al.* 2014). The necessity of using severely immunocompromised mice as host animals severely limits the investigation of such therapies in PDTX models. Likewise, the tumour microenvironment in the PDTX model typically lacks patient-matched stromal cells, which can confer resistance to cytotoxic and targeted therapies (Straussman *et al.* 2012). We, and others, have found that human stromal cells are replaced by murine equivalents upon engraftment in the mouse, suggesting that implanted human cells retain the ability to recruit murine accessory cells to their niche (Bruna *et al.* 2016). However, it should be noted that differences exist between ligand repertoires of human and murine fibroblasts (Mestas & Hughes 2004). Clearly, stromal architecture and activity are mimicked in the murine host; however, it is currently unclear how this reflects the human TME with regards to supporting tumour growth and development.

The advantages of the PDTX over traditional BC models have been discussed extensively both here and elsewhere (Siolas & Hannon 2013, Whittle *et al.* 2015). However, as with any model system, we must be clear of their limitations and learn to interpret preclinical findings within this context. For a detailed discussion of the limitation of the PDTX model, we direct the reader to a prior review by the authors Cassidy *et al.* (2015). Although the tumour’s stromal compartments are undoubtedly important in drug response, the most important factor is undoubtedly the composition of the cancer cell autonomous compartment. Thus, PDTX models are likely the most powerful models currently available in the BC drug discovery process, and every effort should be made to adapt their use to high-throughput screens.

Recently, a PDTX-based drug screening program of unprecedented scale was reported (Gao *et al.* 2015). A large collection (*n* = 1075) of molecularly annotated PDTXs derived from the most common adult cancer types was shown to capture the genomic and transcriptomic features of tumours seen in the clinical population as a whole. The majority of PDTXs in this collection were treated with a variety of targeted compounds in a strategy dubbed ‘1 × 1 × 1’ for ‘one animal per model per treatment’. This approach mimics the reality of human clinical trials, which do not allow for technical or biological replicates. One of the key findings of this study was that a population of PDTXs mimicked the spectrum of human clinical responses, reinforcing the translatability of these models to predict population-based drug responses. Moreover, known mechanisms of resistance were identified by this strategy; for example, three PDTXs treated with encorafenib developed resistance through *BRAF* amplification – a clinically relevant resistance mechanism (Shi *et al.* 2014). It follows that there is a strong rationale for performing drug screens in PDTX models to investigate population-based treatment responses.

## Strategies for breast cancer drug discovery

**Figure 1:**
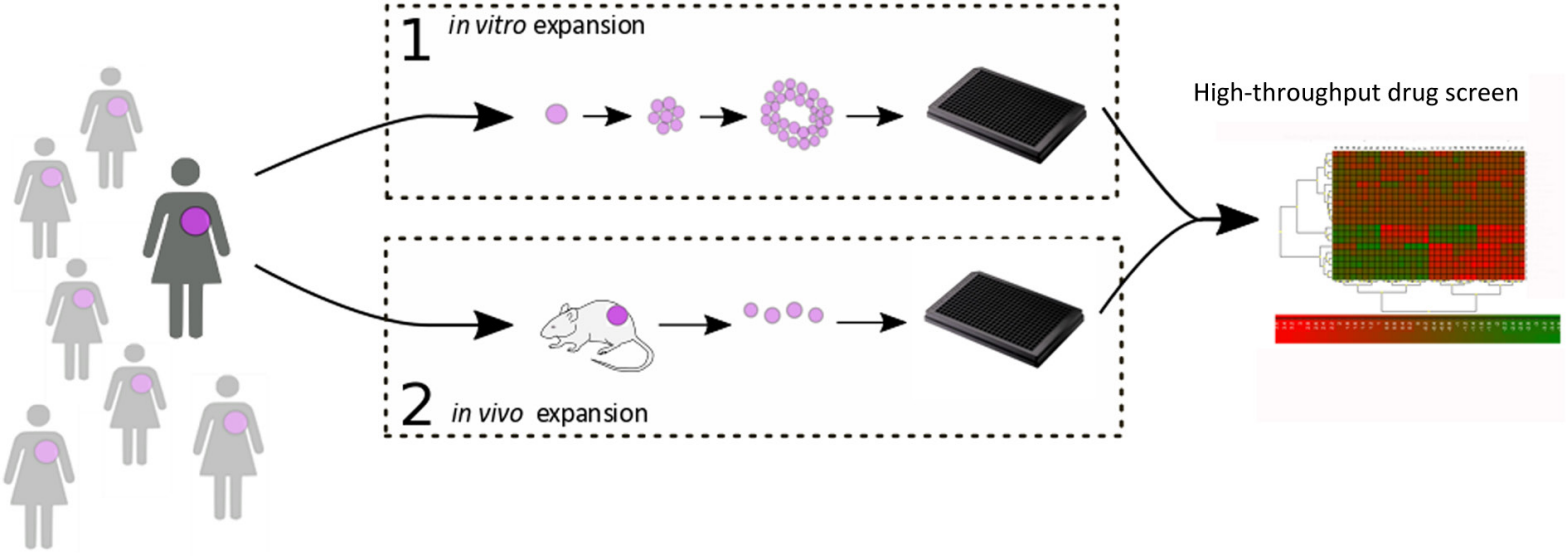
High-throughput drug screen using patient-derived material. Figure 1 highlights high-throughput screening approaches using patient tumour material. (1) represents *in vitro* culture of tumour explants (for example as organoids/tumoroids). (2) represents the integrated PDTX:PDTC platform developed by our lab. In this strategy, patient tumour material is passaged and maintained in the murine host, and patient-derived tumour cells (PDTCs) are periodically dissociated for short-term *ex vivo* culture and high-throughput drug screens.

Despite increasing knowledge of the molecular structure of target proteins and a corresponding increase in the ability to design candidate therapeutics *in silico*, HTS are still essential in the drug discovery process. In order to reduce variability in HTS results, isogenic preclinical models have long been preferred in this regard. However, we have seen in the previous section how such *in vitro* models are not the most faithful reflections of the biological reality, and several steps can be taken to incorporate patient-derived material into this process (Fig. 1). In this section, we consider the impact of tumour heterogeneity on treatment response and explore at which points PDTX models may be introduced into the drug discovery process.

### Tumour heterogeneity

Complex patterns of *inter-* and *intra-*tumour hetero­geneity are a defining feature of human malignancy. Although the past few years have seen an unprecedented increase in research into tumour heterogeneity, we are far from a complete understanding. Indeed, current opinions suggest that cancer is better seen as community of co-existing and co-operating cells than a disease of a specific cell type (Tabassum & Polyak 2015). The origins of this heterogeneity are diverse, but Darwinian evolution of clonal populations has seen the most research in the context of treatment response. Moreover, a varying degree of intratumour heterogeneity exists in triple-negative BCs at diagnosis (Shah *et al.* 2012). Aside from the prognostic features of specific rare subclones (Diaz *et al.* 2012), there is an association between clonal diversity and treatment resistance for at least some tumour types – notably ovarian (Bashashati *et al.* 2013) and oesophageal (Maley *et al.* 2006). Basal-like TNBCs have previously been linked with shorter disease-free survival compared with non-basal-like TNBCs and tend to be associated with a higher clonal diversity (Shah *et al.* 2012, Pereira *et al.* 2016). Together with the clinical observation that targeted therapies often fail to show a sustained and complete response (Aparicio & Caldas 2013), it is apparent that intratumour heterogeneity plays a crucial role in treatment responses. Recently, spatial and temporal heterogeneity, with a mark remodelling of tumour clonal architecture, has been observed in response to aromatase inhibition in BC tumours (Miller *et al.* 2016). Thus, for a more effective oncological drug development process, we should ensure that preclinical models preserve the clonal architecture of the originating tumour sample, and second, that combinatorial drug treatment regimens are investigated to simultaneously target multiple clonal populations and extend time to recurrence.

In the context of targeting multiple specific clonal populations within a tumour, the use of patient-derived preclinical models retaining cancer’s polyclonal architecture is essential. Several groups have attempted to define the clonal structure within PDTX models through mutational clustering by population- and single cell-based computational approaches (Fischer *et al.* 2014, Eirew *et al.* 2015). We have recently quantified heterogeneity in a large biobank of BC PDTXs using statistical tools such as the MATHs score (Mroz & Rocco 2013) and PyClone (Roth *et al.* 2014). PDTXs display a range of heterogeneity similar to that found in the clinical population. PDTXs also preserve most of the originating sample’s intratumour clonal architecture (Eirew *et al.* 2015, Bruna *et al*. 2016). Clonal changes occur to some extent, which were more prominent upon initial engraftment into the mouse than serial passaging, yet these rarely contained breast cancer driver genes (Bruna *et al.* 2016, Pereira *et al*. 2016). In line with previous hypothesis that specific genetic alterations act as markers of fitness and drive evolutionary trajectories, we observed that clonal dynamics are replicated in different mice concurrently engrafted with spatially separated biopsies of the same sample (Eirew *et al.* 2015, Bruna *et al.* 2016). These features position PDTXs as the only preclinical models, currently available, which are able to mimic the intratumour heterogeneity found in the cancer of origin (Cassidy *et al.* 2015). However, further studies are needed to unravel whether the patterns of clonal trajectories seen in PDTX models resemble those seen in the patient.

### Combination drug screens

Resistance to therapy can occur because of differing sensitivity to targeted agents or differing reliance on oncogenic signalling pathways between clonal populations of the same tumour. Although it is often the case that resistant populations exist in a tumour before treatment, acquisition of new mechanisms of resistance can occur as a consequence of tumour evolution. For example, Hata and coworkers have recently shown that acquired resistance of epidermal growth factor receptor (EGFR)-mutant non-small-cell lung cancers to anti-EGFR therapy can occur through the genetic evolution of initially EGFR^T790M^-negative drug-tolerant cells (Hata *et al.* 2016). These *de novo* mutant cells had a distinct phenotype from pre-existing resistant EGFR^T790M^ populations, and drug sensitivity could be restored by co-treatment with Navitoclax, a BCL-2 inhibitor. Thus, tumours are dynamic, constantly evolving populations, and successful combination therapies may be hard to predict from single genomic characterisation.

In a large PDTX study referenced earlier (see the ‘Patient-derived tumour xenografts’ section above), combination therapy resulted in longer disease-free survival compared with single agent therapy (Gao *et al.* 2015). A good example of this is the combination of CDK4/6 inhibitor (LEE011) and PI3K inhibitor (BYL179) in BC models, which showed increased efficacy relative to single-agent treatment. Similar results were seen in melanoma PDTXs with LEE011 and Encorafenib. Although the benefits of using combination therapy to reduce proliferation and apoptosis are well explored, another advantage is delaying the onset of drug resistance. Significantly, some PDTXs treated with LEE011 and Encorafenib combination failed to develop resistance under continuous treatment for up to 200 days. In another combination study conducted by Xu and coworkers, a synergistic effect was observed with MK-8869 and MK-2206 (mTOR and AKT inhibitor, respectively) in two BC PDTXs with high levels of AKT phosphorylation and loss of PTEN expression. MK-2206 was shown to inhibit AKT activation induced by MK-8869, suggesting that combination therapy is essential in tumours with cross-talking signalling pathways (Xu *et al.* 2013). These and other studies strengthen the opinion that drug combinations are essential for more efficacious treatments.

Because PDTX models of BC preserve the clonal architecture of the originating tumour sample, they are greatly suited to study drug–drug combination screens. Although known co-operating signalling pathways (such as MET amplification and EGFR mutation in non-small-cell lung cancer) (Bhang *et al.* 2015) may be simulated in cell line models, the use of PDTX models that mimic human cancer’s complexity and dynamics ensures that our ability to identify novel, clinically relevant, targeted strategies is maximised.

### Predictive biomarker identification

Recent work has highlighted the differential prognostic outcome of mutations in the same gene between patients belonging to different integrative subtypes (Pereira *et al.* 2016). The complex intertumour heterogeneity seen in BC means that drug screens are unlikely to identify novel therapies effective across all patients. Thus, it is essential that we develop large biobanks of PDTX models and deeply characterise them across genomic, transcriptomic and epigenomic spaces. It has been suggested that PDTX models are derived for each patient seen in the clinic and that these personalised PDTX models could be used to inform treatment decisions on a case-by-case basis (Malaney *et al.* 2014). However, this view needs to be taken with caution as there are still many unanswered questions that require further investigation. In the interim, efforts should be focussed on developing robust predictive biomarkers of both drug sensitivity and resistance to extrapolate results from a limited number of models to the BC population as a whole. It is our view that this stratified approach to treatment will be more effective in the near term.

Single-gene mutations are perhaps most widely adopted as clinical biomarkers of response (Garnett *et al.* 2012). As an example, mutations in *EGFR*, *FLT3* and *PIK3CA* predict to some extent the efficacy of targeted therapies directed against the respective mutant proteins (O’Farrell *et al.* 2003, Lynch *et al.* 2004). In other instances, single-gene biomarkers can give insight into the interplay between the drug’s mechanism of action and the tumour’s genetic makeup. For example, *TP53* is an important mediator of apoptosis and cell cycle arrest through its protein product p53. Inactivation of *TP53* through mutation confers resistance to Nutlin-3a, an inhibitor of the MDM2 E3-ligase, which negatively regulates p53 protein levels (Vassilev *et al.* 2004, Garnett *et al.* 2012). Similarly, *RB1* loss and high p16Ink4a levels confer resistance to Palbociclib, a selective CDK4/6 inhibitor showing promising clinical results in ER + BC (O’Leary *et al.* 2016). Mutations in the ligand-binding domain of ESR1 itself have been found in several cases of metastatic ER + BC after treatment with antiestrogen therapy (Robinson *et al.* 2013, Toy *et al.* 2013), but Fulvestrant is potentially effective in such ESR1-mutant cells (Li *et al.* 2013, Merenbakh-Lamin *et al.* 2013, Toy *et al.* 2013). Clearly in cases such as these, biomarkers of drug response consisting of single mutational events are relatively easily understood and implication in the clinic can be swift. Unfortunately, single-gene drug response associations are uncommon, suggesting complex molecular circuits underlie sensitivity to therapy.

Poly(adenosine diphosphate-ribose) polymerase (PARP) inhibitors in ovarian cancer patients have provided one of the best examples to date of stratification based on biomarkers of drug response. PARP inhibitors exert their cytotoxic effect by modulating the repair of DNA damage (Lord & Ashworth 2012). Although originally developed as chemosensitisers, these compounds induce synthetic lethality in tumour cells from patients carrying germline loss-of-function mutations in DNA damage pathway tumour suppressor genes *BRCA1* and *BRCA2* (Lord & Ashworth 2012). Several evidence suggests that the benefit of PARP inhibitors is not restricted to germline *BRCA1/2* mutants and could be associated with a wider range of disrupted mechanisms. Hence, efforts have focussed on establishing a refined *BRCA*ness signature (molecular signatures that mimic the phenotype seen in a *BRCA1/2* germline loss-of-function mutation context) that can better identify patients who would benefit from PARP inhibition treatments (Turner *et al.* 2004, Konstantinopoulos *et al.* 2010).

**Figure 2:**
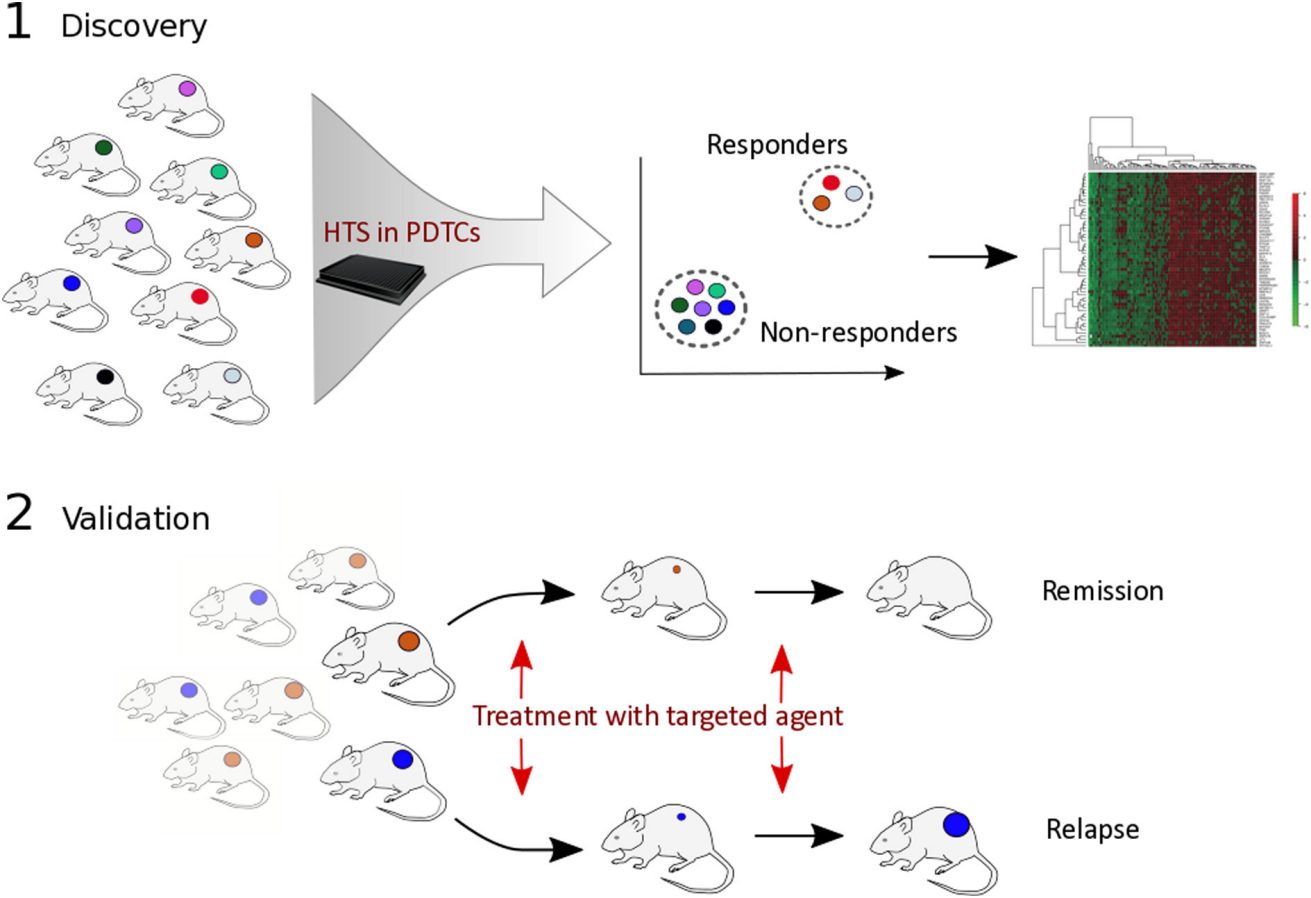
Biomarker discovery using PDTX models. Figure 2 highlights an unbiased approach for biomarker discovery. (1) a mixed cohort of PDTX models is screened with multiple compounds affecting different members of the same signalling pathway and are subsequently clustered based on responders and non-responders. Genomic correlates of drug response are computed before validation *in vivo* (2).

Identification of complex genomic/epigenomic correlates of drug sensitivity requires unbiased and extensive genomic profiling of resistant and sensitive models (Fig. 2). For example, by applying elastic net regression (Zou & Hastie 2005) to genomewide expression data, Garnett and coworkers were able to identify cooperative interactions associated with drug resistance in the NCI60 cell line panel (Garnett *et al.* 2012). Interestingly, the authors observed several instances where transcriptional features correlated with drug sensitivity more than mutational events. For example, Lapatinib (an EGFR/ERBB2 inhibitor for HER2-positive BC) sensitivity unsurprisingly correlated with *ERBB2* expression and mutation status, but the strongest correlate was expression of the matrix metalloproteinase *MMP28.* Likewise, together with *BRAF* mutation, sensitivity to RAF or MEK1/2 inhibitors was recurrently associated with 67 features, including expression of the MAP kinase signalling regulators *SPRY2* and *DUSP4/6* (Hanafusa *et al.* 2002, Garnett *et al.* 2012).

Clearly, biomarkers of treatment response and the emergence of resistance are essential to relate the findings from a limited biobank of PDTX models to the BC population as a whole. This becomes vastly more difficult when attempting to systematically identify correlates of sensitivity from combination drug screens across models that are themselves dynamic, heterogeneous and constantly evolving. Thus, it is essential that PDTX biobanks are characterised and annotated across genomic, epigenomic, transcriptomic and proteomic spaces and that bioinformatics pipelines are incredibly robust and reproducible across labs and through time.

## An integrated pharmacogenomic platform for drug discovery

The previous two sections have described the key features of an ideal BC drug screening program. Over the last 5 years, our lab has developed a successful, robust and reliable framework for the generation of breast cancer explants annotated with extensive molecular data (Bruna *et al.* 2016). Each model has been extensively characterised across genomic, transcriptomic and epigenomic spaces showing that these resemble the originating cancer. The extensive data generated represents a valuable resource to the scientific community, which can be easily browsed using a purpose-built web portal (http://caldaslab.cruk.cam.ac.uk/bcape).

During this time, we have refined our tumour engraftment protocol and now have a large biobank of BC PDTX samples that comprises almost equal percentages of originating primary and metastatic tumour samples. Modifications in the implantation protocol have included, but are not limited to dissociation of tumour samples to single cells with or without *in vitro* culture before implantation and alteration in implantation substrate. Interestingly, speed of implantation after collection in theatre (30–180 min) has been perhaps our most important protocol refinement to date, translating into significantly increased engraftment efficiencies and consequently a more thorough representation of all BC subtypes across the BC PDTX biobank, although with a bias towards ER-negative and poorer prognosis ER-positive tumours (Bruna *et al.* 2016).

A significant limitation of PDTXs as a preclinical platform is the fact that *in vivo* studies are not well suited for high-throughput drug screening due to financial and animal welfare reasons. We have recently reported a method for isolating single-cell suspensions from PDTX tumours for short-term cultured assays (Bruna *et al.* 2016). These PDTX cells (or PDTCs) resemble the originating sample across genomic, epigenomic and transcriptomic landscapes and show similar intratumour clonal architectures. This PDTX/PDTC platform ensures the maintenance of the human BC *in vivo* in the mouse, while allowing for *ex vivo* short-term cultures for single and combination HTS. As a proof of principle, a selection of 22 PDTXs were screened for over 100 different compounds relevant to cancer treatment and the observed drug responses highly correlated across technical and biological replicates. Similarly, compounds with similar target specificities or mechanism of action also had similar responses across all models tested. For example, similar responses were observed in 14 out of the 19 models tested with PARP inhibitor BMN-673 and Cisplatin (a DNA cross-linking agent), both of which exert their effects by increasing the frequency of mis-repaired double-strand breaks in the absence of effective homologous recombination. Also, inhibitors of the PI3K-AKT-mTOR pathway shared a similar pattern of response across all samples tested. To extend these observations, we computed the correlation scores of drug responses for all pair of compounds affecting the same pathway and observed that most compounds with overlapping specificity had similar responses, supporting the biological robustness of our data (Bruna *et al.* 2016). Recognising the importance of combination therapies in achieving long-lasting responses (Friedman *et al.* 2015), we also tested and validated the PDTX/PDTC platform in a HT combinatorial drug screen with standard-of-care chemotherapeutic agents (Cisplatin and Paclitaxel) and six clinically relevant BC-targeted compounds. Remarkably, 33 of 40 (82.5%) drug responses tested *ex vivo* in PDTCs were recapitulated *in vivo* using PDTXs. We propose the use of the PDTX/PDTC platform as a resourceful intermediate in the drug discovery process before *in vivo* testing using PDTXs (Bruna *et al.* 2016).

Finally, to extrapolate our data to the BC population as a whole and to aid in patient stratification during therapy, we sought to identify biomarkers of resistance and response in our models. Analysis of the multidimensional data generated for each model was performed to identify known and novel biomarkers of drug response. Significantly, the PDTX/PDTC platform could identify known mechanisms of drug response and resistance. However, the heterogenous nature of plausible biomarkers of drug response spread across the models tested suggests that integrated genomic data will be a stronger predictor of drug response than single-gene biomarkers.

It is our belief that the integrated pharmacogenomics PDTX/PDTC platform will show the greatest utility in preclinical studies and will considerably shorten the time of testing new drugs in patients (Bruna *et al.* 2016). This approach extends the predictive value of PDTX models in oncological drug development by allowing HT screening of hundreds of compounds, as well as reducing considerably the financial costs and number of animals used in such studies.

## Conclusions and future directions

The oncological drug space suffers from an unsustainable rate of attrition, in part attributed to preclinical models that fail to accurately represent the complexity of BC. BC PDTX models broadly maintain the heterogeneity of their originating patient tumour; however, historically they have suffered from considerable engraftment bias towards TNBCs. Although the lower-fidelity BC models (cell lines etc.) have long been used in the drug discovery process, the inherent low throughput of PDTX models has also limited their adoption. In the context of clonal diversity and intertumour heterogeneity, it is essential that we adapt the models best reflecting their originating sample to the drug discovery process. Here, we have considered BC models currently used in the drug discovery process and reflected on our own experiences in adapting BC PDTX models to HTS in our integrated PDTX/PDTC platform (Bruna *et al.* 2016). Efforts such as these can only be achieved if the establishment of PDTX models is shared across large collaborative networks such as the EuroPDX consortium (Hidalgo *et al.* 2014) or in the context of large pharmaceutical companies. Alternatively, the process of establishing and maintaining large cohorts of PDTX models might be best suited to pharmaceutical companies (Gao *et al.* 2015).

The current generation of PDTX models is unlikely to be their final iteration. It is important to stress areas where improvements can be made and emphasise that no single model system can represent fully the complexity of a human malignancy. Ideally, the next generation of PDTX models would incorporate tumour extrinsic compartments of the microenvironment specific to the patient, either in the form of matched patient peripheral blood leukocytes or matched patient fibroblasts. These humanised PDTX models (huPDTX) would significantly increase the utility of the model and perhaps decrease the selective pressures seen on engraftment. However, the generation of huPDTX models is fraught with difficulties, and technical challenges are yet to be overcome (Cassidy *et al.* 2015). An intermediate solution to screen targeted therapies with an essential immune component could be to isolate and expand patient-derived peripheral blood leukocytes and incorporate them into complex short-term co-cultures with PDTCs. The use of checkpoint inhibitors in BC is of paramount interest (Ali *et al.* 2015) and as such this strategy could be of substantial utility in future drug screens.

## Footnote

This paper is part of a thematic review section on hormone-dependent cancers. The Guest Editor for this section was Wayne Tilley.

## Declaration of interest

The authors declare that there is no conflict of interest that could be perceived as prejudicing the impartiality of this review.

## Funding

This work was supported by Cancer Research UK (grant number RG84936).

## Author contribution statement

J W C and A B wrote the manuscript. A S B and W G contributed to structure, content and editing of the manuscript. All authors approved the final version of the manuscript.
